# Application of metagenomic next-generation sequencing technology in the etiological diagnosis of peritoneal dialysis-associated peritonitis

**DOI:** 10.1515/biol-2022-0865

**Published:** 2024-04-26

**Authors:** Shan-Shan Guo, Gang Fu, Yan-Wei Hu, Jing Liu, Yu-Zhu Wang

**Affiliations:** The Nephrology Department, Beijing Haidian Hospital, Haidian District, Beijing 100191, China; The Nephrology Department, Beijing Haidian Hospital, No. 29 Zhongguancun Street, Haidian District, Beijing 100191, China

**Keywords:** metagenomic next-generation sequencing, blood culture flask method, peritoneal dialysis-associated peritonitis, infection, pathogen

## Abstract

Pathogens detected by metagenomic next-generation sequencing (mNGS) and the laboratory blood culture flask method were compared to understand the advantages and clinical significance of mNGS assays in the etiological diagnosis of peritoneal dialysis-associated peritonitis (PDAP). The study involved a total of 37 patients from the hospital’s peritoneal dialysis centre, six of whom were patients with non-peritoneal dialysis-associated peritonitis. Peritoneal dialysis samples were collected from the 37 patients, who were divided into two groups. One group’s samples were cultured using conventional blood culture flasks, and the other samples underwent pathogen testing using mNGS. The results showed that the positive rate of mNGS was 96.77%, while that of the blood culture flask method was 70.97% (*p* < 0.05). A total of 29 pathogens were detected by mNGS, namely 24 bacteria, one fungus, and four viruses. A total of 10 pathogens were detected using the bacterial blood culture method, namely nine bacteria and one fungus. The final judgment of the PDAP’s causative pathogenic microorganism was made by combining the clinical condition, response to therapy, and the whole-genome sequencing findings. For mNGS, the sensitivity was 96.77%, the specificity was 83.33%, the positive predictive value was 96.77%, and the negative predictive value was 83.33%. For the blood culture flask method, the sensitivity was 70.97%, the specificity was 100%, the positive predictive value was 100%, and the negative predictive value was 0%. In conclusion, mNGS had a shorter detection time for diagnosing peritoneal dialysis-related peritonitis pathogens, with a higher positive rate than traditional bacterial cultures, providing significant advantages in diagnosing rare pathogens.

## 1 Introduction

Peritoneal dialysis-associated peritonitis (PDAP) is a common and serious complication of peritoneal dialysis, affecting its therapeutic effect and threatening patients’ lives [1]. The occurrence of PDAP is related to various factors, such as non-standard fluid replacement according to aseptic principles, peritoneal dialysis catheter-related infections, decreased intestinal function leading to the displacement of intestinal bacteria, and reduced peritoneal barrier function caused by long-term dialysis. Moreover, high-risk operations that may cause peritonitis are becoming more common [2]. For the treatment of PDAP, the application of multiple antibiotics active against Gram-positive and Gram-negative bacteria often occurs before the pathogen is determined, even though the targeted application of an antibiotic once a pathogen is known is more likely to result in success [3]. The blood culture flask method is currently the main method of detecting pathogenic bacteria. Although it can be accurate, this approach has shortcomings, such as a long culture time and generally low culture-positive rates [4]. Failure to identify pathogenic bacteria in many patients with PDAP has led to the empiric application of multiple antibiotics, increasing the toxic side effects of antibiotics and affecting the cure rate of peritonitis.

With the development of technology, especially the advancement and commercialisation of genetic technology, the price of testing is decreasing, making clinical genetic sequencing possible. The term metagenome, also known as a microbial environmental genome, was proposed by Handelsman et al. in 1998 to refer to the sum of all the biological genetic material in the environment, including genes for culturable and nonculturable microorganisms [5]. Metagenomic next-generation sequencing (mNGS) is a culture-free, non-preferred pathogen detection technique based on next-generation sequencing technology. High-throughput sequencing of deoxyribonucleic acid (DNA) and ribonucleic acid (RNA) extracted directly from clinical samples, followed by database alignment and bioinformatics analysis, allows for the detection of bacteria, fungi, viruses, parasites and other pathogens simultaneously [6]. Recently, mNGS has made many advances in identifying novel, rare, and important pathogens [7]. The range of mNGS assays covers 6,350 bacteria, 1,798 DNA viruses, 1,064 fungi, and 234 parasites whose current genome sequences are known. mNGS identifies the genus and species of pathogenic bacteria, classifies types, and indicates drug resistance genes; this is of great significance to providing clinical anti-infection treatment and conducting epidemiological investigation [8]. Currently, mNGS plays an important role in the diagnosis of various clinical diseases, including sepsis [9], alveolar inflammation [10], postoperative infection [11], respiratory disease diagnosis [12], and infection caused by immunodeficiency viruses [13]. However, there are no relevant reports of mNGS in diagnosing PDAP pathogens.

This study compared the sensitivity, specificity, positive and negative predictive values of the blood culture and mNGS methods for detecting PDAP, hoping to identify a more accurate and rapid diagnostic tool to provide a more precise clinical treatment based on analysis results.

## 2 Materials and methods

### 2.1 Study participants

Clinical data and ascites samples were collected between 1 January 2021 and 31 August 2022 from 37 patients with long-term attendance at the hospital’s peritoneal dialysis centre. Six patients were diagnosed with non-PDAP; 31 patients met the diagnostic criteria for PDAP established by the International Society for Peritoneal Dialysis (ISPD) in 2016 [14]. The results of 37 samples analysed by conventional bacterial culture and mNGS were submitted. This study was approved by the Medical Ethics Committee of the Beijing Haidian Hospital; the ethical approval number is BHHMEC-XM-2021-15. All the patients signed an informed consent form before each mNGS test.


**Informed consent:** Informed consent has been obtained from all individuals included in this study.
**Ethical approval:** The research related to human use has complied with all the relevant national regulations, and institutional policies and by the tenets of the Helsinki Declaration, and has been approved by the author’s institutional review board.

### 2.2 Inclusion and exclusion criteria

The inclusion criteria were as follows: (a) patients with PDAP diagnosed according to the 2016 ISPD criteria [14]; namely patients with (1) abdominal pain and cloudy peritoneal dialysate, with or without fever and other symptoms; (2) a leukocyte count of >100 μL and polymorphonuclear cells >50% in the peritoneal dialysate; and (3) a positive dialysis pathogen smear or culture. Diagnosing PDAP required meeting at least two of the criteria; and (b) patients that were followed up with long-term peritoneal dialysis.

The exclusion criteria were as follows: (a) patients with incomplete clinical data and (b) patients with systemic infection.

### 2.3 Study methods

Peritoneal dialysate of 100 mL was extracted from the 37 patients with a sterile, nucleic acid-free syringe in strict accordance with the aseptic principle of the peritoneal dialysate abdomen dwell time being >2 h. Of this extract, 10 mL of peritoneal dialysate was used for the ascites routine and 40 mL for the two groups of ascites culture using blood culture flasks. The remaining 50 mL of peritoneal dialysate was placed into a nucleic acid-free sampling tube and submitted to the Gene Sequencing Corporation. Culture and identification were performed in a microbiology laboratory.

Blood culture flask method: Peritoneal dialysis solution was injected aseptically into blood culture flasks (standard aerobic and anaerobic flasks); the flasks were then placed into the instrument incubator of the supporting BacT/ALERT 3D Microbial Detection System (bioMérieux France, Craponne, France). When a positive culture flask alarm was indicated, a sterile syringe was used to draw a 40 µL culture medium for microbial smears. Two culture medium plates, with blood agar and MacConkey agar, were simultaneously inoculated and incubated at 35℃. Cultures incubated for 7 days were considered negative if there was no bacterial growth detected. For positive blood cultures, microbiological analysis of the growing pathogens was identified using the VITEK 2 Compact Microbial Analysis System (bioMérieux France, Craponne, France).

mNGS: 50 mL of the samples were taken, centrifuged at 7,800 rpm for 10 min for 1 mL of precipitate, and DNA and RNA were extracted. The DNA extraction was performed using a PathoXtract^®^ Basic universal kit (WYXM03211S, Willingmed, Beijing, China), and the extraction was performed using a PathoXtract^®^ Virus DNA/RNA coextraction kit (WYXM03009S, Willingmed, Beijing, China) according to the kit instructions. The DNA and RNA were then mixed, and the RNA was reverse transcribed using a SuperScript™ duplex copy DNA (cDNA) synthesis kit (11917020, Invitrogen, Waltham, MA, USA) to obtain the cDNA for subsequent library construction.

Subsequently, the merged DNA library was constructed using an Illumina^®^ DNA Prep (M) Tagmentation (20018705, Illumina, Inc., San Diego, CA, USA) kit according to the kit’s instructions. Sequencing was performed with a single-end 75 bp sequence on a NextSeq™ 550Dx (Illumina, Inc., San Diego, CA, USA). No less than 20 million pieces of data were obtained for each sample. The negative control samples were synchronised in each batch for quality control.

Sequencing data were automatically analysed to obtain detection reports. Among them, the pathogenic microbial genome data of bacteria, fungi, viruses, parasites and archaea were obtained from the National Centre for Biotechnology Information GenBank^®^ database, and a clinical application-level reference genome database was constructed following the steps of genome filtering, screening, and validation. High-quality sequencing data were aligned to the human reference genome GRCh37 (hg19) using alignment software to remove human host sequences and obtain clean data that could be used for subsequent identification of pathogenic microorganisms. The clean data were compared with the established pathogen microbial database to complete the information annotation of the pathogen microbial species information, to provide the sequence number, relative abundance, and genome coverage of the species and to complete the reference report.

The judgment methods used for the mNGS results were as follows:

Bacteria (excluding mycobacteria), viruses, and parasites: the threshold for a bacterial diagnosis was a number of sequences greater than 30. If the result was a single bacterium or a highly pathogenic pathogen, it could still be identified as the pathogenic organism, even if the number of sequences was small.

Fungi: the diagnostic threshold was an abundance of the genus greater than 15% and a number of sequences greater than 50.

Mycobacterium: When the number of measured sequences of mycobacteria was ≥1, the reading was considered positive.

Viruses: Given that the presence of viruses in the peritoneal dialysate of non-peritonitis patients is rare, the possibility of viral colonisation was high [15].

### 2.4 Identification of the pathogenic microorganism causing PDAP

The final identification of the pathogenic microorganism causing PDAP required a combination of the clinical presentation, microbial characteristics, and disease outcome results [14]. The following assumptions were made:

1. If mNGS and the laboratory blood culture flask methods identified the same microorganism, it was assumed to be the final pathogenic microorganism.
2. If mNGS identified a variety of microorganisms, then the microorganism with the most sequences (usually five times that of any other microorganism) was likely to be the pathogenic microorganism.
3. If a single microorganism was identified by mNGS and the number of sequences was larger than the reference value of environmental contamination, it was regarded as the pathogenic microorganism.
4. If the detected microorganism was highly pathogenic, even if it was not an organism that normally colonised the abdominal cavity, it was judged as a pathogenic microorganism based on the clinical presentation.

### 2.5 Statistical analysis

Data processing and analysis were performed with the SPSS® (Statistical Software™ Version 22.0, IBM SPSS Statistics, IL, USA). Enumeration data were output as a relative digital composition (%) or ratio (%) using Fisher’s exact test. The sensitivity (number of true positives/[number of true positives + number of false negatives]), specificity (number of true negatives/[number of true negatives + number of false positives]), positive predictive value (number of true positive cases/[number of true positive cases + number of false positive cases]) and negative predictive value (number of true negative cases/[number of true negative cases + number of false negative cases]) of the two pathogen determination methods were calculated. Kappa values were used to compare the consistency of the mNGS results with the clinical microbial evidence. Statistical significance was considered to be *p* < 0.05.

## 3 Results

### 3.1 Basic patient information

**Figure 1:**
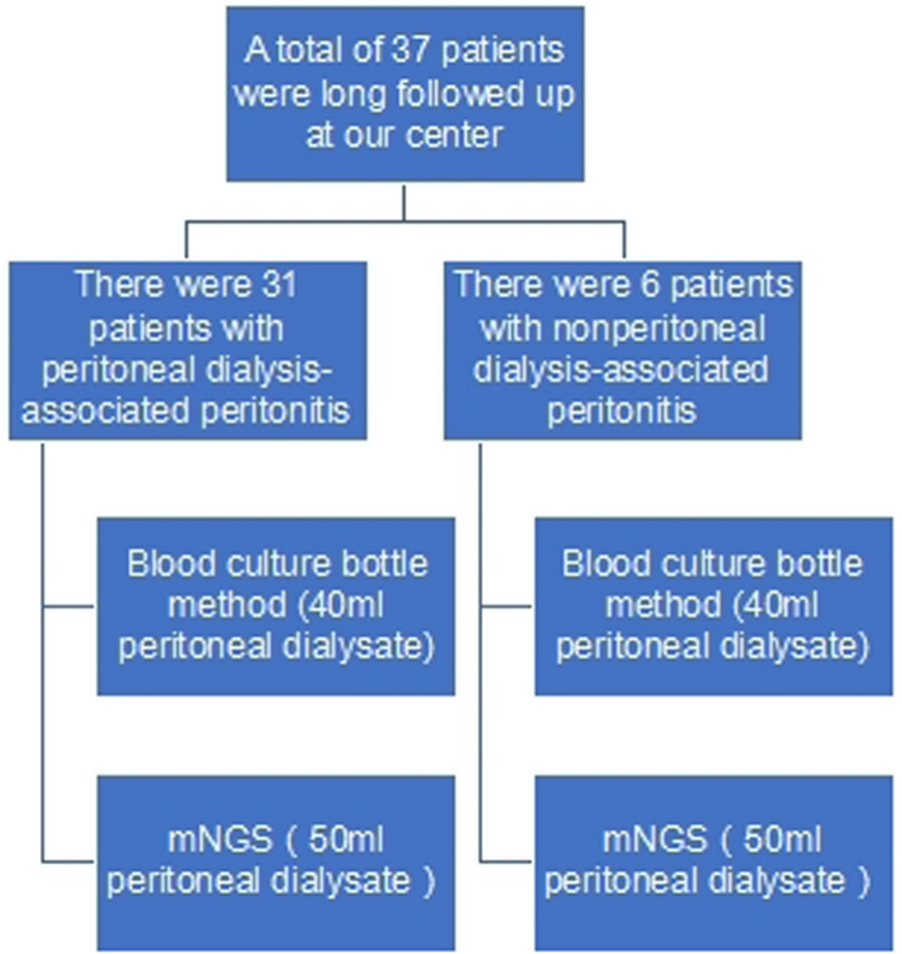
Flow chart.

The 37 patients, 25 of whom were men and 12 women, were aged 36–82 (61.05 ± 13.04 years old). The primary cause of uraemia was as follows: there were 22 patients with diabetic nephropathy; six patients with chronic nephritis; three patients with hypertensive renal injury; two patients with urinary tract obstruction; one patient with polycystic kidney disease; and three patients with unknown causes (Table 1 and Table S1). All the mNGS analyses were completed within 24 h, and the traditional laboratory tests took 1–15 days (Figure 1). Cases 20 and 29 eventually died, eight patients were switched to haemodialysis, and the remaining 20 patients improved significantly.

**Table 1:** Basic characteristics of the patients

	*N*/ \[\bar{X}]\] ± *S*
Gender	
Male	25
Female	12
Age (year)	61.05 ± 13.04
Causes	
Diabetic nephropathy	22
Chronic nephritis	6
Hypertensive renal injury	3
Urinary tract obstruction	2
Polycystic kidney disease	1
Unknown causes	3
Total	37

### 3.2 Pathogen distribution

In this study, 37 samples were cultured in duplicate blood culture flasks; the samples were simultaneously submitted for mNGS. A total of 29 pathogens were detected by mNGS and consisted of 24 bacteria, one fungus, and four viruses. The top three identified pathogenic organisms were *Staphylococcus* (17.86%), *Escherichia coli* (16.07%), and *Klebsiella pneumoniae* (8.92% [Table 2]). A total of 10 pathogens, of which nine were bacteria and one fungus, were detected by the blood culture flask method. The top three species identified as pathogenic were *Staphylococcus* (28.57%), *E. coli* (28.57%), and *K. pneumoniae* (14.28% [Table 2]).

**Table 2:** Pathogen distribution (n/Detection rate)

Pathogen classification	mNGS	Blood culture
*Staphylococcus haemolyticus*	4/0.071	3/0.143
*Enterococcus pallidum*	1/0.018	—
*Klebsiella pneumoniae*	5/0.089	3/0.143
*Alcaligenes faecalis*	1/0.018	—
*Morganella morganii*	2/0.036	—
*Escherichia coli*	9/0.160	6/0.286
*Enterococcus hirae*	1/0.018	—
*Staphylococcus aureus*	1/0.018	—
*Streptococcus salivarius*	1/0.018	—
*Serratia marcescens*	1/0.018	1/0.048
*Enterococcus faecalis*	1/0.018	—
*Acinetobacter berezonii*	1/0.018	—
*Mycobacterium tuberculosis*	2/0.036	—
*Gardnerella vaginalis*	1/0.018	—
*Staphylococcus epidermidis*	4/0.071	3/0.143
*Leuconostoc*	1/0.018	—
*Candida parapsilosis*	1/0.018	1/0.048
*Enterobacter cholerae*	1/0.018	—
*Leuconostoc lactis*	1/0.018	1/0.048
*Motibacter putrefaciens*	1/0.018	—
*Streptococcus pneumoniae*	1/0.018	—
*Klebsiella aerogenes*	1/0.018	—
*HPgV Virus Type C*	3/0.054	—
*Human herpesvirus 5*	5/0.089	—
*Human herpesvirus 6A*	2/0.036	—
*Corynebacterium unicolor*	1/0.018	—
*Prevotella melaninogenica*	1/0.018	—
*Streptococcus panorale*	1/0.018	—
*Human metapneumovirus*	1/0.018	—
*Leuconostoc mesenteroides*	—	1/0.048
*Streptococcus sanguinis*	—	1/0.048
*Enterococcus* spp.	—	1/0.048

Note: mNGS:metagenomic next-generation sequencing.

### 3.3 Sensitivity, specificity, positive and negative predictive values of the mNGS

To make the final judgment concerning the identity of the pathogenic microorganism, a combination of clinical presentation, microbial characteristics, and disease outcome was used as the gold standard. The positive detection rate of mNGS was 96.77% compared with 70.97% for the blood culture flask method. The positive rate of mNGS was significantly higher than that of the blood culture method (*p* < 0.05). The sensitivity of mNGS was 96.77%, the specificity was 83.33%, the positive predictive value was 96.77% and the negative predictive value was 83.33%. The sensitivity of the blood culture flask method was 70.97%, the specificity was 100%, the positive predictive value was 100%, and the negative predictive value was 40% (Tables 3 and 4). Furthermore, the positive rate for mNGS was 93.3 and 73.3% for the bacteria culture (*p* < 0.05). The results demonstrated mNGS’s high positive detection rate and sensitivity.

**Table 3:** Sensitivity, specificity, positive predictive values, and negative predictive values of Blood culture flask method

		Pathogenic bacteria determined by clinical diagnosis	
		Positive	Negative
Blood culture flask method	Positive	22	0
	Negative	9	6

Note: sensitivity 70.97%; specialty 100%; Positive Predictive Value (PPV) = 100%; Negative Predictive Value (NPV) = 40%.

**Table 4:** Sensitivity, specificity, positive predictive values, and negative predictive values of mNGS

		Pathogenic bacteria determined by clinical diagnosis	
		Positive	Negative
mNGS	Positive	30	1
	Negative	1	5

Note: sensitivity, 96.77%; specialty, 83.33%; Positive Predictive Value (PPV) = 96.77%; Negative Predictive Value (NPV) = 83.33%; mNGS, metagenomic next-generation sequencing.

## 4 Discussion

In recent years, the incidence of peritonitis has declined, but PDAP remains an important cause of dialysis treatment failure and can endanger patients’ lives [1]. Identifying the type of pathogen and achieving targeted application of antibiotic therapy is vital to successful PDAP treatment. Currently, the double blood culture flask method is the primary method of detecting pathogenic bacteria. According to ISPD peritoneal dialysis-related peritonitis guidelines [14], the positive pathogen culture analysis rate is required to reach 80%, but only a few centres can achieve this. In recent years, several literature reports have shown that the positive rate of pathogen culture in PDAP has increased to 61–72.2%; however, this figure still does not meet the required standard. The present study’s blood culture flask method positive rate was 70.97%, which was consistent with previously reported results, but the figure continues to fall short of the desired goal. Although traditional bacterial culture methods are accurate, the culture time is protracted, with it usually taking between 1 and 15 days to obtain results. Moreover, the bacterial culture-positive rate is generally low. The reasons for the low positive rate of pathogen culture are as follows: (1) some patients have used antibiotics before going to the hospital; (2) clinically, unqualified operation for obtaining peritoneal dialysate sample, and the bacterial content in the abdominal dialysate may be too small; and (3) bacterial culture conditions are harsh and easily affected by a variety of factors, such as the culture temperature, culture media, PH value, humidity and the pathogen’s ease of culture. With the development of molecular technologies, new techniques, such as polymerase chain reaction (PCR) and quantitative bacterial DNA PCR, have been used to detect peritonitis pathogens. However, PCR is used for the validation of relevant bacteria by designing primer probes and numerous basic experiments, which is demanding for bioinformatics analyses. Therefore, there has been little clinical application [16]. However, with the advancement of science and technology, genetic testing has become more mature and commercialisable, making mNGS in clinical settings possible. Li et al. [10] reported that a patient developed symptoms of pneumonia, but no pathogen was found after three biopsies. With the help of mNGS, the pathogen causing the patient’s external skin dermatitis was finally diagnosed as *Dermatitis bacteriophage*. Qu et al. [11] reported that mNGS is more sensitive than routine pathogen testing in diagnosing paediatric haematopoietic stem cell transplantation infection and can contribute to a clinical diagnosis. The mNGS of cerebrospinal fluid samples reported by Gan et al. [17] was used as a complementary test for cryptococcal meningeal diagnosis and enabled clear differentiation between *Cryptococcus gatti* and *Cryptococcus neoformans*.

This present study showed that the sensitivity of mNGS was 96.77%, the specificity was 83.33%, the positive predictive value was 96.77%, and the negative predictive value was 83.33%. The sensitivity of the blood culture flask method was 70.97%, the specificity was 100%, the positive predictive value was 0% and the negative predictive value was 40%. These results demonstrated that mNGS is much more sensitive than the blood culture flask method and pathogens are easier to detect, which is similar to the results of previous studies [9,10,11]. The differences between mNGS and the blood culture flask methods may be related to the different emphasis of each detection method. Blood culture focuses on detecting the pathogen function, and only some bacteria and fungi can be detected. Furthermore, some pathogens are difficult to culture or cannot be cultured at all using this method. Another possible reason is that the medium encourages the growth of a dominant bacterium during blood culture, and a competitive relationship develops between the dominant bacterium and any other bacteria present.

In contrast, mNGS focuses on pathogenic microorganism species, with the detection results covering a wide range of organisms. Although mNGS is more sensitive than blood culture, the specificity is lower, possibly because mNGS can detect both intact pathogenic viable bacteria and non-pathogenic nucleic acid fragments. This suggests that pathogenic bacteria identification should use a combination of clinical presentation, microbial characteristics, and disease outcome data.

Despite its benefits, false negatives can occur with mNGS. First, false negatives may arise because pathogen loads are below the detection depth. This depth can vary and ranges from a short 75 bp sequence to a 10,000 bp long sequence; the more sequences collected, the higher the sensitivity. Second, there are excessive background gene sequences in the host and microecology. The literature indicates that over 99% of metagenomic sequences are human and microecological gene sequences, with pathogenic microorganisms accounting for less than 1% [18]. Third, microbes have thick cell walls. For example, obtaining *Mycobacterium tuberculosis* nucleic acid sequence*s* via mNGS is difficult, and wall-breaking techniques are needed. Moreover, it is proposed in the literature that changes in host gene transcription [19], spreading [20] of sequence distribution of pathogenic nucleic acid, and microecological diversity change [21] help to distinguish between infection, colonisation, contamination, and background bacteria, and these issues are increasingly the focus of mNGS research.

Although mNGS has advantages over blood culture methods, it has some limitations in clinical use. First, it is expensive compared to traditional culture methods and unsuitable for universal detection in all patients with suspected infectious diseases [22]. Second, each test facility needs consistent quality control; otherwise, its results can be inaccurate. Most nucleic acids found in pathogenic samples have a human background. Over 90% of sequencing data typically comes from human sequences, reducing pathogen sensitivity detection. Therefore, it is necessary to efficiently remove nucleic acids from the human host background to improve the sensitivity of pathogen detection [23]. Currently, there are no uniform interpretation criteria for mNGS reports. Since mNGS detects nucleic acid fragments, it can be performed if the nucleic acid fragments present in the sample can achieve valid detection data. In addition, it is impossible to distinguish if the reported organisms have colonised or infected or if the infection is previous or current. Therefore, NT infection needs to be identified using a combination of clinical manifestations and physical and chemical results. Thresholds, relative abundance, sequence readout, genome coverage, and depth are commonly used reference indicators in clinical practice; however, there is no uniform standard [24,25]. Li et al. [24] determined the infectious pathogen threshold by using mNGS analysis for a lung tissue biopsy of a suspected contagious disease, and the results were compared with standard cultures as follows: r-polar abundance at the bacterial or fungal genus level was >30%; sequence reads of a single species of bacteria or fungi in positive samples by culture or histopathological examination was ≥50; and sequence reads for *Mycobacterium* were *t* ≥ 1. There is no uniform standard for threshold, relative abundance, or sequence reads of mNGS. Therefore, mNGS needs further research and development.

The present study had several limitations. First, this study was a single-centre study with a small number of peritoneal dialysis-related peritonitis cases, so the sample size requirements for diagnostic tests could not be fully met, and there was a possibility of result bias. To draw more reliable conclusions will require large-scale multi-centre studies. Second, increased follow-up time is required for patients since the short review period may have affected the results. Third, the PDAP infections with positive mNGS pathogen identification that were not supported by traditional culture method results need further verification by PCR testing. Combined with this study’s results, the authors believe that the threshold of mNGS test results should be adjusted according to the actual situation, with the number of bacterial pathogen sequences commonly used clinically being >20, as the bacterial diagnostic threshold and the number of common fungal pathogen sequences in clinical practice are >50 as the diagnostic threshold. For some difficult-to-culture pathogens, such as *M. tuberculosis*, the infections they cause could be considered when their sequence is ≥1. When conventional bacterial cultures are negative, even the low sequence number of detected pathogens should be considered as pathogens. For example, in the present study, patient 26 was negative for blood culture flask analysis; however, the mNGS was positive for *Streptococcus pneumoniae,* and the sequence number was 1. The importance of identifying the pathogen is reflected in the fact that the patient’s condition improved after treatment with antibiotics effective against *S. pneumoniae*.

## 5 Conclusion

In conclusion, mNGS is a rapid, efficient, and accurate technique for detecting pathogenic microorganisms. Despite its limitations, the technique has excellent value in the pathogen identification of peritoneal dialysis-associated systemic inflammation and can be used as a complementary method to conventional culture methods.

## Supplementary Material

Supplementary Table
