# Supplementary material for: Application of metagenomic next-generation sequencing technology in the etiological diagnosis of peritoneal dialysis-associated peritonitis
Source: Open Life Sci. 2024 Apr 26;19(1):20220865. doi: 10.1515/biol-2022-0865 (PMC11049737; doi:10.1515/biol-2022-0865)
Supplement: Supplementary Table [file biol-2022-0865-sm.pdf]

Supplementary material

Table S1: Basic information of 37 patients

Number	Gender	Yea (years)	Results of blood culture flask method	mNGS results	Clinical final judgment
1	Male	66	*Staphylococcus haemolyticus*	*Staphylococcus haemolyticus 845*<br>*HPgV virus type C 5*<br>*Human Herpesvirus 5 (cytomegalovirus) 5*	*Staphylococcus haemolyticus*
2	Male	76	*Enterococcus*	*Enterococcus neoformans 8*	*Enterococcus neoformans*
3	Male	82	*Bacteria Klebsiella pneumoniae* Subspecies	*Klebsiella pneumoniae 312*<br>*Alcaligenes faecalis 62*<br>*Morganella morganii 23*<br>*Escherichia coli 17*<br>*Enterococcus hirae 3*	*Bacteria Klebsiella pneumoniae* Subspecies
4	Female	49	*Escherichia coli*	*Escherichia coli 3586*	*Escherichia coli*
5	Male	82	Negative	*Staphylococcus aureus 24*	*Staphylococcus aureus*
6	Male	75	*Streptococcus sanguinis*	*Streptococcus salivarius 214*<br>*Human herpes virus 6A 44*	*Streptococcus salivarius*
7	Male	58	*Serratia marcescens*	*Serratia marcescens 359*<br>*Enterococcus faecalis 41*<br>*Acinetobacter baumannii 14*	*Serratia marcescens*
8	Female	61	Negative	*Mycobacterium tuberculosis complex 3024*	*Mycobacterium tuberculosis*
9	Male	60	*Staphylococcus haemolyticus*	*Staphylococcus haemolyticus 6602*	*Staphylococcus haemolyticus*
10	Female	36	Negative	*Gardnerella vaginalis 20*	*Gardnerella vaginalis*
11	Male	37	*Staphylococcus epidermidis*	*Staphylococcus epidermidis 824*	*Staphylococcus epidermidis*
12	Male	50	*Leuconostoc lactis*	*Leuconostoc bacteria395*	*Leuconostoc lactis*
13	Male	41	*Candida parapsilosis*	*Candida parapsilosis 5857*	*Candida parapsilosis*
14	Female	62	Negative	*Enterobacter hormaechei 31*<br>*Klebsiella Pneumoniae 20*	*Enterobacter hormaechei*
15	Male	57	Negative	*Morganella morganii 29*<br>*Escherichia coli 9*	*Morganella morganii*
16	Male	50	*Leuconostoc pseudomesenteroides*	*Staphylococcus epidermidis 121*<br>*Leuconostoc lactis 24*	*Staphylococcus epidermidis*<br>*Leuconostoc pseudomesenteroides*
17	Female	80	*Escherichia coli*	*Escherichia coli 312235*<br>*Human herpes virus 5 (cytomegalo virus) 35*<br>*HPgV virus C 1*	*Escherichia coli*

(Continued)

Table S1: *Continued*

Number	Gender	Yea (years)	Results of blood culture flask method	mNGS results	Clinical final judgment
18	Male	56	*Bacteria Klebsiella pneumoniae* Subspecies	*Klebsiella pneumoniae 132962*<br>*Human herpes virus 6A 78*	*Bacteria Klebsiella pneumoniae* Subspecies
19	Female	74	Negative	*Mycobacterium tuberculosis complex 970*	*Mycobacterium tuberculosis*
20	Female	46	*Bacteria Klebsiella pneumoniae* Subspecies	*Klebsiella pneumoniae 731*	*Bacteria Klebsiella pneumoniae* Subspecies
21	Male	66	*Staphylococcus epidermidis*	*Staphylococcus epidermidis 24388*<br>*Acinetobacter pittii 170*<br>*Human herpes virus 5189*<br>*HpgV virus C 66*	*Staphylococcus epidermidis*
22	Female	55	*Escherichia coli*	*Escherichia coli 148857*	*Escherichia coli*
23	Female	67	*Escherichia coli*	*Escherichia coli 209925*	*Escherichia coli*
24	Female	76	*Escherichia coli*	*Escherichia coli 6151*	*Escherichia coli*
25	Male	67	*Staphylococcus epidermidis*	*Staphylococcus epidermidis 894*	*Staphylococcus epidermidis*
26	Male	54	Negative	*Streptococcus pneumoniae 1*	*Streptococcus pneumoniae*
27	Female	78	*Escherichia coli*	*Escherichia coli 304191*<br>*Klebsiella aerogenes 4064*	*Escherichia coli*
28	Male	69	*Staphylococcus haemolyticus*	*Staphylococcus haemolyticus 23288*<br>*Human herpes virus 531*	*Staphylococcus haemolyticus*
29	Male	69	*Staphylococcus haemolyticus*	*Staphylococcus haemolyticus 1019*	*Staphylococcus haemolyticus*
30	Male	58	Negative	*Staphylococcus hominis 162*<br>*Corynebacterium 85*<br>*Human herpes virus 531*	*Staphylococcus hominis*
31	Male	40	Negative	Negative	Unknown
32	Female	69	Negative	Negative	None
33	Male	77	Negative	*human metapneumovirus 47*<br>*Prevotella melaninogenica 46 Human microecology*<br>*Oral streptococcus 36 Human microecology*	None
34	Male	51	Negative	Negative	None
35	Male	65	Negative	*Human herpes virus 531*	None
36	Male	45	Negative	*Human herpes virus 5554*<br>*Human herpes virus 6A3*	None
37	Male	55	Negative	Negative	None

Note: mNGS: metagenomic next-generation sequencing.
